# High-density lipoprotein cholesterol is a predictor of survival in cirrhotic patients with acute gastrointestinal bleeding: a retrospective study

**DOI:** 10.1186/s12876-020-01522-6

**Published:** 2020-11-16

**Authors:** Ran Cheng, Ning Tan, Qian Kang, Hao Luo, Hongyu Chen, Jiali Pan, Yifan Han, Yuqing Yang, Xiaoyuan Xu

**Affiliations:** Department of Infectious Diseases, Peking University First Hospital, 8 Xishiku Street, Beijing, 100034 China

**Keywords:** High-density lipoprotein cholesterol, Liver cirrhosis, Acute gastrointestinal bleeding, 6-Week mortality

## Abstract

**Background:**

Lipid profiles are declined in patients with viral liver cirrhosis and correlated with severity of liver disease. Hepatitis B virus (HBV) is the leading cause of liver cirrhosis in China. Our primary aim was to investigate whether serum lipids and lipoproteins associate with survival in patients with HBV-related cirrhosis and acute gastrointestinal bleeding, and develop a 6-week mortality risk score that incorporates it.

**Methods:**

From January 2008 to December 2015, consecutive cirrhotic patients with acute gastrointestinal bleeding admitted to our hospital were evaluated and randomly divided into the derivation (n = 629) and validation (n = 314) cohorts. A logistic regression model was established to confirm the association between lipoprotein cholesterol and mortality. Accuracy to predict mortality were assessed by area under the receiver operating characteristic curves (AUROCs) and compared using the Hanley and McNeil test.

**Results:**

Among study subjects, the 6-week mortality rate was 10.6%. High-density lipoprotein cholesterol (HDL-C) level was found to correlate most strongly with prognostic scores. On ROC analysis, HDL-C showed excellent diagnostic accuracy for 6-week mortality. Logistic regression analysis provided a simple algorithm based on the combined use of 4 variables (total bilirubin (TBIL), HDL-C, International normalized ratio, and hemoglobin), allowing accurate discrimination of 3 distinct prognostic subgroups with 1.7% (low risk), 12.3% (intermediate risk), and 56.9% (high risk) mortality. Its accuracy was significantly better than that of Child–Pugh, model of end-stage liver disease, albumin-bilirubin score, D’Amico model, Augustin model, AIMS65 score and Glasgow-Blatchford score. Baseline HDL-C values ≤ 0.54 mmol/L were associated with markedly lower 6-week survival. Comparable results were found in the validation set.

**Conclusion:**

HDL-C is a potential indicator for the prognosis of patients with cirrhosis and acute gastrointestinal bleeding. The new algorithm based on HDL-C allowed an accurate predictive assessment of 6-week mortality after bleeding attack.

## Background

Liver cirrhosis is an important cause of morbidity and mortality as a consequence of continuous liver injuries [1]. Acute gastrointestinal bleeding is a serious complication and critical clinical event in cirrhotic patients [2]. Despite the significant improvements in general therapeutic management of critically ill patients and in specific haemostatic therapy, the mortality rate remains high, ranging from 6 to 20% in different studies [3]. The factors associated with the striking difference in life expectancy may include the severity of complications and the degree of liver failure [4, 5]. Conventional prognostic scores such as Child–Pugh [6], model of end-stage liver disease (MELD) [7] and albumin-bilirubin score (ALBI) [8] have been useful for assessing the prognosis of general patients with liver cirrhosis, but their predictive performances remain suboptimal for the estimation of outcomes after an episode of acute gastrointestinal bleeding in patients with cirrhosis. D’Amico score [9] and Augustin model [10] was designed especially for acute variceal bleeding (AVB) and complicated to calculate. Scoring systems for gastrointestinal bleeding like AIMS65 score [11] and Glasgow-Blatchford score [12] are not specific for patients with cirrhosis in whom the severity of liver dysfunction is closely associated with patients’ outcomes. Therefore, identification of objective indicator obtained from routine examinations that can provide a predictive value is warranted.

Since the liver plays a crucial role in cholesterol homeostasis [13], hypocholesterolemia often occurs in patients with chronic liver diseases [14]. The decrease in serum levels of lipids and lipoproteins is highly prevalent in cirrhotic patients, with a prevalence that increases in parallel with the disease severity [15]. Previous studies have shown that high-density lipoprotein cholesterol (HDL-C) was an independent predictor of mortality in cirrhotic patients [16] and associated with the severity of sepsis [17]. However, studies regarding the effects of liver disease on lipid profiles have reached conflicting conclusions. The reason for the discrepancy could be the different etiology. Chronic hepatitis B is the leading cause of liver cirrhosis in China. To eliminate the influence of the etiology, the present study was focus on hepatitis B virus (HBV)-related cirrhotic patients. We aimed to investigate the prognostic value of lipid profiles in cirrhosis and acute gastrointestinal bleeding, and develop a simple and practical risk model to better predict 6-week mortality incorporates it.

## Methods

### Patients

From January 2008 to December 2015, 943 patients with HBV-related cirrhosis and acute gastrointestinal bleeding admitted to Peking University First Hospital, were evaluated and randomly divided into the derivation (n = 629) and validation (n = 314) cohorts. Liver cirrhosis was confirmed either by liver biopsy or by clinical presentations, routine liver function tests and medical imaging techniques. Acute upper gastrointestinal bleeding was considered in patients presenting with hematemesis, melena, and/or nasogastric aspirate of fresh or dark blood drained by a nasogastric tube. The exclusion criteria were as follows: (1) other causes of chronic liver disease; (2) the use of lipid-regulating drugs within half a year; (3) terminal illness involving the major organs, such as severe heart failure, chronic obstructive pulmonary disease, and malignancy other than hepatocellular carcinoma (HCC); (4) lack of complete medical records; (5) previous liver transplantation; (6) pregnancy. The study was performed in line with the 1975 Declaration of Helsinki and was approved by the Ethics Committee of Peking University First Hospital, Beijing, China. (Approval No. 20161126).

Patients received standard management at our institution that included continuous proton pump inhibitor infusion, vasoactive drugs for 5 days, and prophylactic parenteral antibiotics upon admission. Packed red blood cell transfusions (PRBC) were given to achieve a target hemoglobin level over 70 g/L. Unless contraindications to endoscopy existed, all patients with upper gastrointestinal bleeding underwent endoscopy performed by an experienced endoscopist as soon as safely possible. Sclerotherapy or ligation choice was left to the endoscopist’s discretion according to recommended standards. Transjugular intrahepatic portosystemic shunt (TIPS) was used for variceal bleeding that could not be controlled by endoscopy. Treatment of nonvariceal bleeding (NVB) was left to the discretion of the endoscopist.

### Clinical data collection and prognostic scores

The parameters collected at admission included demographic data, medical comorbidities, medication use, vital signs, laboratory tests and electrocardiogram (ECG). Endoscopy findings and outcomes were recorded. Routine biochemical tests were performed in our hospital laboratory.

QT length was assessed by an observer blinded to patients. The QT interval was corrected for heart rate according to the ‘cirrhosis-specific’ formula [18] (QTc = QT/RR1/3.02) in cirrhotic patients. The prolongation of the QTc interval was defined as a length > 450 ms in men and > 470 ms in women.

Prognostic scores including the Child–Pugh [6], MELD [7], ALBI [8], D’Amico score [9], Augustin model [10], AIMS65 score [11], and Glasgow-Blatchford score [12] were calculated and graded at admission using the formulas from the original article (Table 1).

**Table 1:** Prognostic models evaluated

Model	Equation		
Child–Pugh score [6]	Calculated from five variables including bilirubin, albumin, prothrombin, ascites status and degree of encephalopathy		
MELD score [7]	9.57 × ln (creatinine mg/dl) + 3.78 × ln (bilirubin mg/dl) + 11.20 × ln (INR) + 6.43		
ALBI score [8]	− 0.085 × (albumin g/L) + 0.66 × lg (bilirubin μmol/L)		
D’Amico model [9]	− 0.083 + 0.88 × hepatic encephalopathy (no = 1, mild = 2, severe = 3) + 0.22 × (bilirubin mg/dl) + 1.12 × HCC (yes = 1, no = 0) – 0.86 × (albumin g/dl)		
Augustin model [10]	– 7.226 + 0.475 × Child–Pugh score + 1.746 × HCC (yes = 1, no = 0) + 1.179 × (creatinine ≥ 1 mg/dl, yes = 1, no = 0)		
N-GIB score	0.005 × TBIL(mmol/L) – 0.112 × Na(mmol/L) – 2.757 × HDL (mmol/L) – 0.017 × HGB (g/L) + 16.121		
AIMS65 [11]	Score	Glasgow-Blatchford score [12]	
Albumin < 3 mg/dL	1	Risk factor	Score
INR > 1.5	1	BUN, mg/dL	
Altered mental status	1	≥ 18.2 to < 22.4	2
Systolic BP < 90 mmHg	1	≥ 22.4 to < 28.0	3
Age > 65 years	1	≥ 28.0 to < 70.0	4
		≥ 70.0	6
		Hemoglobin, men g/dL	
		≥ 12.0 to < 13.0	1
		≥ 10.0 to < 12.0	3
		< 10.0	6
		Hemoglobin, women g/dL	
		≥ 10.0 to < 12.0	1
		< 10.0	6
		Systolic BP, mm Hg	
		100–109	1
		90–99	2
		< 90	3
		Other markers	
		Pulse ≥ 100 (bpm)	1
		Presentation with melena	1
		Presentation with syncope	2
		Hepatic disease	2
		Cardiac failure	2

MELD score was calculated according to the United Network for Organ Sharing modification: The minimum value for the laboratory parameters within the formula was set to 1. The maximum value for serum creatinine was set to 4. BP, blood pressure; bpm, beats per minute; HCC, hepatocellular carcinoma; HDL, high-density lipoprotein cholesterol; HGB, Hemoglobin; INR, international normalized ratio

### Statistical analysis

Data were analyzed using SPSS (version 20.0, IBM, USA) and MedCalc software (version 18.2.1, Ostend, Belgium). Statistically significant differences were indicated by a two-sided P-value < 0.05 for all analyses. Continuous variables with a normal distribution were reported as the mean ± SD and non-normal variables were presented as medians and interquartile ranges (IQRs). Categorical variables are expressed as frequencies (percentages). Differences between derivation and validation cohorts were compared by the independent samples t-test for continuous variables and the χ^2^ test for categorical variables. The correlation was evaluated by Pearson test. The predicting performance for mortality was assessed by area under the receiver operating characteristic curve (AUROC) and compared using the Hanley and McNeil test [19]. Cut-off values were obtained with the maximal Youden index (sensitivity + specificity-1) [20]. Univariate logistic regression was performed to assess the association between potential risk factors and mortality. Variables with *P* values < 0.05 in univariate analysis were used for multiple logistic regression analysis. The Hosmer–Lemeshow goodness-of-fit statistic was used to test the reliability of the model [21]. Variables that maintained *P* < 0.05 in multiple logistic regression analysis of the derivation cohort were weighted according to the odds ratio (OR) and 95% confidence intervals (CI) to develop a scoring system for predicting mortality. Survival and death risk analyses were performed using the Kaplan–Meier method and differences between groups were assessed by the log-rank test. In the present study, the threshold to define high risk was set at a mortality rate of 20% [10], and low risk was set at 10% [22].

## Results

### Patient characteristics

A total of 943 HBV-related cirrhosis and acute gastrointestinal bleeding were included, of whom 629 and 314 patients were randomly enrolled into the derivation and validation cohorts, respectively. Table 1 compares demographic, clinical, and laboratory characteristics between the two cohorts of patients. The two cohorts were not entirely matched: in the derivation cohort, the percentage of PRBC transfusion was lower and endoscopic treatment was higher than those among the patients in the validation cohort (Table 2).

**Table 2:** Baseline characteristics and outcomes of the derivation cohort and validation cohort

Parameters	Derivation cohort (n = 629)	Validation cohort (n = 314)	*P* value
Age, years	55 (14)	53 (14)	0.37
Male sex	484 (76.9%)	243 (77.3%)	0.88
BMI	24.16 (3.92)	23.88 (3.78)	0.30
Systolic blood pressure (mmHg)	115 (22)	112 (25)	0.31
Cirrhosis etiology			
Hepatitis B virus n (%)	629 (100%)	314 (100%)	1.00
Variceal bleeding, n (%)	369 (58.7%)	167 (53.2%)	0.11
Heart rate	78 (21)	80 (22)	0.81
Hemoglobin, g/L	97 (46)	99 (49.25)	0.72
Total leukocyte count, × 10^9^/L	3.71 (1.8)	3.9 (2.85)	0.61
Platelets, × 10^9^/L	66 (52)	73 (56)	0.27
Alanine aminotransferase, IU/L	27 (26)	27 (27.25)	0.83
Creatinine, μmol/L	78 (26.25)	76 (23)	0.06
Sodium, mmol/L	139.8 (5.5)	139.6 (4.93)	0.64
Bilirubin, mmol/L	24 (28.72)	23.6 (26.6)	0.71
Albumin, g/L	31.1 (8.45)	31.5 (9.53)	0.97
Triglyceride	0.73 (0.46)	0.79 (0.51)	0.09
Cholesterol, mmol/L	3.01 (1.31)	3.1 (1.33)	0.32
High density lipoprotein, mmol/L	0.83 (0.49)	0.84 (0.49)	0.58
Low-density lipoprotein, mmol/L	1.63 (0.97)	1.64 (1.05)	0.59
International normalized ratio	1.22 (0.3)	1.23 (0.33)	0.77
Fibrinogen, g/L	1.76 (0.85)	1.78 (0.88)	0.45
Hepatocellular carcinoma	137 (21.7%)	76 (24.2%)	0.40
Previous bleeding, n (%)	203 (32.2%)	119 (35.9%)	0.09
Ascites, n (%)	367 (58.3%)	183 (58.2%)	0.98
Hepatic Encephalopathy, n (%)	56 (8.9%)	22 (7.0%)	0.32
β-blocker use, n (%)	133 (21.1%)	73 (23.2%)	0.46
QTc, ms	435 (33)	433.5 (37)	0.57
QTc prolongation, n (%)	148 (23.5%)	81 (25.7%)	0.44
PRBC transfusion (%)	170 (27%)	105 (27.8%)	0.04
> 2 PRBC transfusion (%)	57 (33.5%)	43 (41.0%)	0.03
Endoscopic treatment, n (%)	260 (41.3%)	153 (40.5%)	0.03
TIPS treatment, n (%)	23 (3.6%)	11 (2.9%)	0.91
6-Week death, n (%)	66 (10.4%)	34 (10.8%)	0.75
Prognostic scores			
Child–Pugh score	8 (4)	8 (4)	0.86
Child–Pugh A	202 (32.1%)	93 (29.6%)	
Child–Pugh B	264 (42%)	141 (44.9%)	
Child–Pugh C	163 (25.9%)	80 (25.5%)	
MELD	10.98 (7.76)	10.8 (7.02)	0.19
ALBI	−1.72 (0.96)	−1.71 (0.99)	0.89
D’Amico model	−1.32 (1.39)	−1.27 (1.4)	0.75
Augustin model	−2.95 (2.29)	−2.95 (2.22)	0.97
Glasgow-Blatchford score	9 (7)	9 (8)	0.74
AIMS65	1 (1)	1 (1)	0.88

*PBRC* packed red blood cells transfused; All values are expressed as median (IQR), or n (%)

### Lipid profiles correlate with liver disease severity

Lipid profiles include triglyceride, cholesterol, HDL-C and low-density lipoprotein cholesterol (LDL-C). In the derivation and validation cohorts, the association of individual lipid fractions with prognostic models (Child–Pugh, MELD, and ALBI) was assessed by Pearson correlation, as shown in Table 3. HDL-C was found to have strongest correlation with liver function scores.

**Table 3:** Correlation between lipid profile levels and prognostic models

Correlation	Triglyceride	Cholesterol	HDL-C	LDL-C
*Derivation cohort*				
Child–Pugh	R = −0.160, *P* = 0.000	R = −0.337, *P* = 0.000	R = −0.458, *P* = 0.000	R = −0.307, *P* = 0.000
MELD	R = −0.061, *P* = 0.129	R = −0.298, *P* = 0.000	R = −0.458, *P* = 0.000	R = −0.263, *P* = 0.000
ALBI	R = −0.168, *P* = 0.000	R = −0.341, *P* = 0.000	R = −0.452, *P* = 0.000	R = −0.318, *P* = 0.000
Validation cohort				
Child–Pugh	R = −0.225, *P* = 0.000	R = −0.300, *P* = 0.000	R = −0.331, *P* = 0.000	R = −0.258, *P* = 0.000
MELD	R = −0.029, *P* = 0.613	R = −0.290, *P* = 0.000	R = −0.442, *P* = 0.000	R = −0.213, *P* = 0.000
ALBI	R = −0.256, *P* = 0.000	R = −0.293, *P* = 0.000	R = −0.369, *P* = 0.000	R = −0.254, *P* = 0.000

### Derivation cohort: predictors of 6-week mortality

On ROC analysis, HDL-C showed excellent diagnostic accuracy for 6-week mortality, with an AUROC of 0.847 (95% CI 0.789–0.905). The best cut-off value of HDL-C was 0.54 mmol/L, with a sensitivity of 85.1% and specificity of 74.2%.

We performed univariate and multivariate analysis to assess the independent predictors of 6-week mortality. Variables included in the univariate analysis are shown in Table 4. The variables significantly associated with 6-week mortality in the univariate analysis were as follows: age, systolic blood pressure, ascites, hepatic encephalopathy, HCC, heart rate, PRBC transfusion, endoscopic treatment, hemoglobin (HGB), total leukocyte count (WBC), alanine aminotransferase (ALT), serum sodium (Na), total bilirubin (TBIL), albumin (ALB), QTc interval prolongation, cholesterol, HDL-C, LDL-C, serum creatinine (Scr), international normalized ratio (INR), fibrinogen (FIB) (Table 4). Multivariate logistic regression analyses showed that TBIL, HDL-C, Na, and HGB were independently associated with 6-week death (Table 4). Based on the multivariate regression coefficients, we calculated a new prognostic model for HBV-related cirrhotic patients with acute GIB named N-CGIB according to the following formula: 0.005 × TBIL (mmol/L) – 0.112 × Na (mmol/L) – 2.757 × HDL (mmol/L) – 0.017 × HGB (g/L) + 16.121. The N-CGIB model showed an excellent discrimination for 6-week death prediction, with an AUROC of 0.895 (95% CI 0.853–0.936). Hosmer and Lemeshow analysis confirmed goodness-of-fit for the model (*P* = 0.117).

**Table 4:** Univariate analyses of predictive factors associated with 6-week mortality (derivation cohort)

Parameters	Univariate	
	Odds ratio (95% CI)	*P* value
Age, years	1.027 (1.003 1.052)	0.028
Male sex	1.021 (0.556 1.875)	0.947
BMI	0.900 (0.829 0.977)	0.012
Systolic blood pressure	0.959 (0.941 0.976)	0.000
Hepatocellular carcinoma	2.647 (1.549 4.522)	0.000
Heart rate	1.038 (1.024 1.052)	0.000
Hemoglobin, g/L	0.985 (0.976 0.994)	0.001
Total leukocyte count, × 10^9^/L	1.171 (1.104 1.242)	0.000
Platelets, × 10^9^/L	1.002 (0.998 1.006)	0.378
Alanine aminotransferase, IU/L	1.002 (1.001 1.004)	0.007
Creatinine, μmol/L	1.002 (1.000 1.004)	0.038
Sodium, mmol/L	0.817 (0.777 0.859)	0.000
Bilirubin, mmol/L	1.011 (1.008 1.014)	0.000
Albumin, g/L	0.893 (0.853 0.935)	0.000
Cholesterol, mmol/L	0.396 (0.293 0.535)	0.000
High-density lipoprotein, mmol/L	0.007 (0.002 0.020)	0.000
Low-density lipoprotein, mmol/L	0.401 (0.266 0.605)	0.000
International normalized ratio	17.48 (7.55 40.471)	0.000
Fibrinogen, g/L	0.448 (0.283 0.708)	0.001
Previous bleeding (%)	1.138 (0.666 1.946)	0.636
Ascites present (%)	3.233 (1.724 6.064)	0.000
Hepatic Encephalopathy present (%)	7.04 (3.784 13.097)	0.000
Endoscopic treatment, n (%)	0.418 (0.233 0.753)	0.004
TIPS treatment, n (%)	1.847 (0.609 5.604)	0.278
PRBC transfusion (%)	2.033 (1.202 3.441)	0.008
> 2 PRBC transfusion (%)	1.248 (0.801 1.591)	0.066
QTc prolongation Male (> 450 ms) Female (> 470 ms)	2.720 (1.604 4.613)	0.000

Parameters	B	SE	Wals	Odds ratio (95% CI)	*P* value
*Multivariate analyses: Predictors of 6-week mortality*					
Bilirubin, mmol/L	0.005	0.002	6.037	1.004 (1.001–1.007)	0.000
High-density lipoprotein, mmol/L	−2.757	0.662	17.331	0.063 (0.017–0.232)	0.000
Sodium, mmol/L	−0.112	0.031	12.851	0.894 (0.840–0.950)	0.000
Hemoglobin, g/L	−0.017	0.006	7.251	0.983 (0.971–0.995)	0.007
Constant	16.121	4.253	14.366	10,031,946.4	0.000

**Fig. 1:**
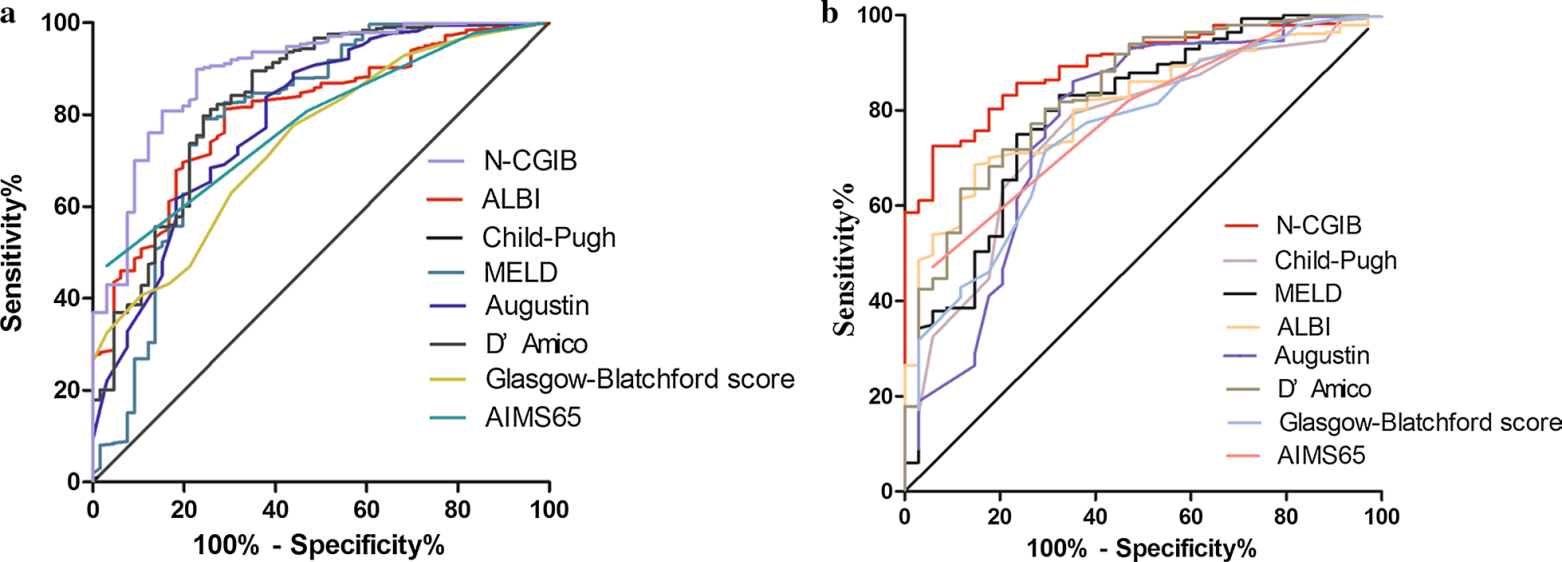
Comparison of predictive performance of N-CGIB score with Child–Pugh, MELD, ALBI, D’Amico, Augustin model, AIMS65 score, and Glasgow-Blatchford score in the derivation cohort (**a**) and validation cohort (**b**)

The developed model showed an excellent predictive accuracy, with AUROCs significantly better than that of Child–Pugh, MELD, ALBI, D’Amico score, Augustin model, AIMS65 score and Glasgow-Blatchford score (Table 5, Fig. 1a).

**Table 5:** Discrimination of the prognostic models in the derivation and validation cohorts

Parameters	AUROC (95% CI)	*P* value	AUROC (95% CI)	*P* value
*Derivation cohort Validation cohort*				
N-CGIB	0.895 (0.853–0.936)		0.893 (0.850–0.937)	
Child–Pugh	0.800 (0.746–0.853)	*P* = 0.000	0.757 (0.673–0.842)	*P* = 0.002
MELD	0.795 (0.724–0.865)	*P* = 0.000	0.797 (0.712–0.881)	*P* = 0.011
ALBI	0.806 (0.756–0.856)	*P* = 0.000	0.808 (0.744–0.872)	*P* = 0.013
D’Amico model	0.835 (0.777–0.893)	*P* = 0.025	0.836 (0.765–0.907)	*P* = 0.108
Augustin model	0.793 (0.731–0.854)	*P* = 0.001	0.766 (0.667–0.866)	*P* = 0.009
AIMS65 score	0.777 (0.727–0.828)	*P* < 0.001	0.773 (0.697–0.849)	*P* = 0.003
Glasgow-Blatchford	0.742 (0.684–0.800)	*P* < 0.001	0.753 (0.669–0.837)	*P* = 0.001

### Mortality in external validation cohort

HDL-C was an excellent predictor of 6-week mortality (AUROC 0.827; 95% CI 0.752–0.901) in the validation cohort.

Hosmer and Lemeshow analysis confirmed the goodness-of-fit of the N-CGIB model (*P* = 0.066). The diagnostic accuracy of the N-CGIB model (AUROC 0.893; 95% CI 0.850–0.937) was similar to D’Amico model (AUROC 0.836; 95% CI 0.765–0.907), significantly better than that of the other models, including Child–Pugh (AUROC 0.757; 95% CI 0.673–0.842), MELD (AUROC 0.797; 95% CI 0.712–0.881), ALBI (AUROC 0.808; 95% CI 0.744–0.872), Augustin model (AUROC 0.766; 95% CI 0.667–0.866), AIMS65 score (AUROC 0.773; 95% CI 0.697–0.849), and Glasgow-Blatchford (AUROC 0.753; 95% CI 0.669–0.837) (Table 5, Fig. 1b).

### Discrimination of N-CGIB for variceal bleeding and non variceal bleeding

We also analyzed the prognostic performance of N-CGIB in the subset with variceal hemorrhage considering the whole group of enrolled patients (536 of 953; 56.2%). As seen, N-CGIB score (AUROC 0.914, 95% CI 0.878–0.951) showed an excellent predictive accuracy for AVB. The discriminatory performance of the N-CGIB model (AUROC 0.841; 95% CI 0.783–0.896) was lower for NVB as compared to that for AVB.

### Survival

In the derivation cohort, 66 of the 629 (10.5%) patients died during study observation, with early death rate (16 of 66, 24.2%) occurring within the first five days. In the test set, 34 of the 314 (10.8%) patients died during study observation, with early death rate (7 of 34, 20.5%) occurring within the first five days.

**Fig. 2:**
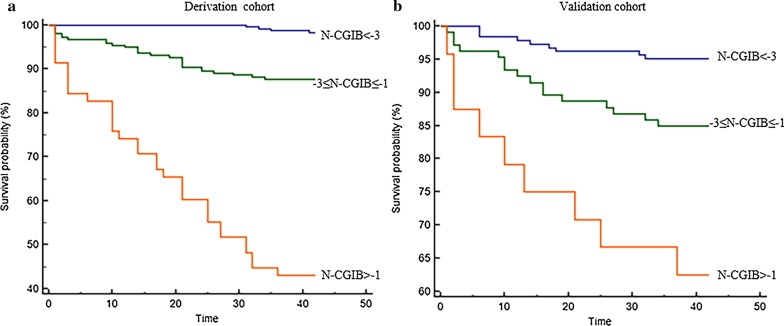
Probability of 6-week survival in cirrhotic patients with acute gastrointestinal bleeding according to the developed model in the derivation and validation sets. N-CGIB identified 3 risk groups: low-risk (< -3), medium-risk (−3 to −1), high-risk (> −1). The probability of survival progressively and significantly decreased across groups, both in the derivation (A) and validation (**b**) sets (*P* < 0.001)

Mortality increased with increasing N-CGIB score. The final selected cutoff value accurately discriminated patients in 3 subgroups with distinct prognosis: a low-risk group (N-CGIB score below -3), whose in-hospital mortality was 1.7%; an intermediate-risk group (N-CGIB score range from -3 to -1), with a 12.3% mortality; and a high-risk group (N-CGIB score more than -1) associated with a 56.9% or greater predicted risk. Comparable results were found in the validation set with a significant decrease in 6-week probability of survival: 1.1%, 17.9%, and 54.2%, respectively. (Fig. 2a, b).

**Fig. 3:**
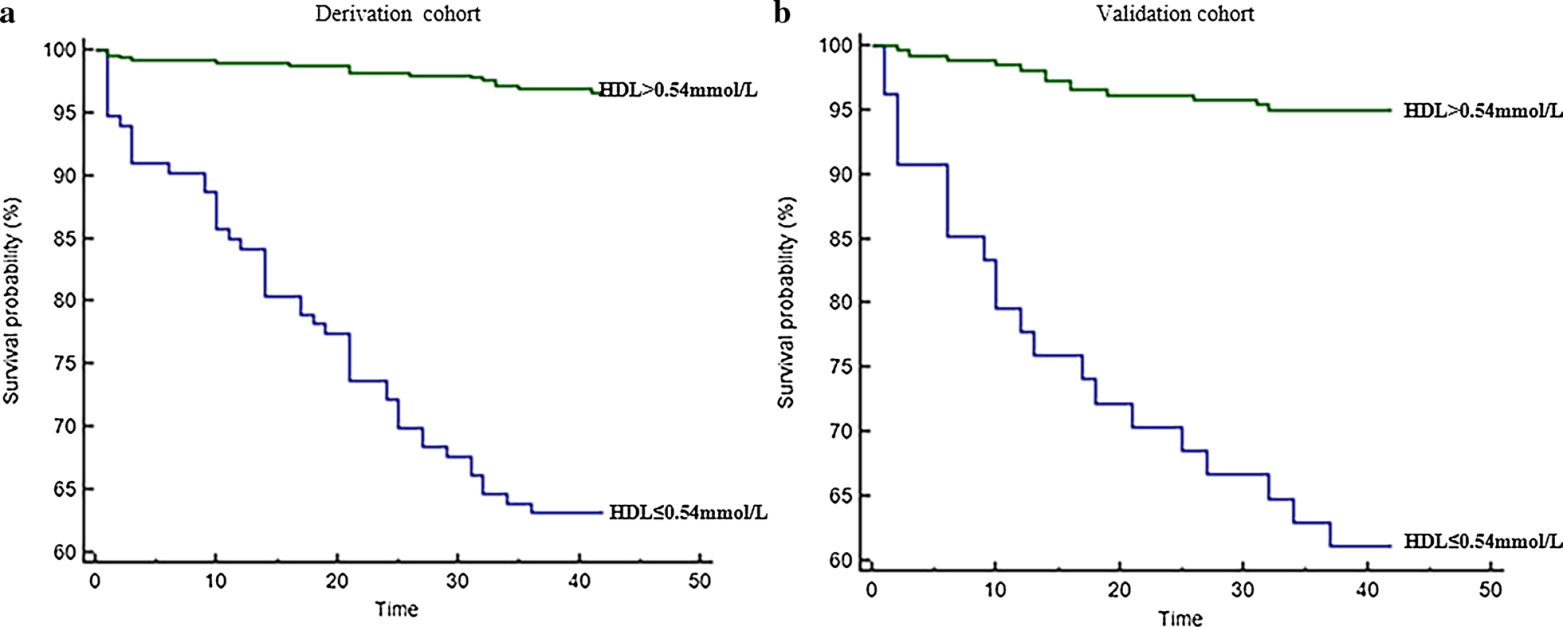
Mortality was significantly higher in patients with HDL-C ≤ 0.54 mmol/L (*P* < 0.001 by log-rank test) in the derivation (**a**) and validation (**b**) sets

On Kaplan–Meier analysis, baseline HDL-C values ≤ 0.54 mmol/L were associated with markedly lower 6-week survival in the derivation cohorts (Fig. 3a), and validation cohorts (Fig. 3b).

## Discussion

Previous studies showed that chronic liver diseases strongly impair the lipid metabolism with the hypercholesterolemia and hyperlipidemia as a common finding in viral hepatitis [23]. Additionally, in patients with viral liver cirrhosis, total serum cholesterol, LDL, and HDL-cholesterol were lower than that in patients with chronic active hepatitis [24]. In this retrospective study, we showed that HDL-C level, cholesterol and LDL-C level, as measured by routine automated laboratory methods, are correlated with validated prognostic indexs (Child–Pugh, MELD, and ALBI). Among them, HDL-C was the most remarkable indicator. On ROC analysis, HDL-C shows excellent diagnostic accuracies for 6-week mortality in patients with HBV-related cirrhosis and acute gastrointestinal hemorrhage, with AUROCs similar to those of composite scores. Using a large patient cohort, we confirmed that HDL-C level was evidently lower in HBV-related cirrhotic patients who died than in survivors. In multivariate analysis, HDL-C is confirmed as an independent indicator of prognosis. Then we finally formulated a risk scoring system named N-CGIB to predict 6-week mortality for HBV-related cirrhotic patients admitted with acute gastrointestinal bleeding. For predicting death, the N-CGIB model performed significantly better than the conventional scoring models. HDL levels less than 0.54 mmol/L were associated with a 13-fold increase in the hazard ratio for 6-week death.

Acute gastrointestinal bleeding is common in cirrhosis and account for significant mortality. In more than 80% of cases death was related to liver failure or to complications derived from hemorrhage, such as bacterial infections, kidney injury or other organ failures associated with decompensated cirrhosis [4, 5]. HDL-C was confirmed as a risk factor of 6-week mortality in the present study. A complex number of factors may contribute to this phenomenon, first, serum HDL-C is considered as a marker of liver function, which further decreased due to hepatocyte ischemia provoked by anemia, tachycardia and arterial hypotension; second, HDL-C has been discovered as a modulator of both intrinsic and extrinsic coagulation cascades in vitro experiments [25].

Our second aim was to devise and validate a prognostic system predicting 6-week mortality risk of bleeding patients. By logistic regression analysis, we develop a simple and practical model based on HDL-C, integrated by 3 other predictors (TBIL, HGB, and Na), which could be acquired upon admission. Prognostic indicators were related to the severity of liver dysfunction and bleeding. Other potentially relevant variables (number of packed red blood cells, endoscopic treatments, or TIPS) failed to show an independent prognostic effect in multivariate analysis. Thus, N-CGIB can inform clinician’s clinical judgment upon admission to the hospital, before endoscopic therapy. For predicting 6-week mortality, the developed model performed significantly better than the previously described models. The Hosmer–Lemeshow test indicated a good prediction performance. It is applicable to acute gastrointestinal hemorrhage regardless of its source.

We categorized N-CGIB based on -3 and -1 thresholds and yielded 3 groups. These cut-off values divided patients into groups with low, medium, and high risk of mortality. Importantly, this stratification confirmed its strength in the validation set. N-CGIB can easily distinguish patients at low risk from those at high risk and allow clinicians to begin assessment of a patient’s mortality risk upon admission to the hospital and allow for appropriate management.

The limitations of this study should be acknowledged. First, this study was a single-center retrospective study. Inevitably, bias could arise from missing data and selection criteria. Thus, the results should be validated in prospective multicenter studies using larger sample sizes. Second, a proportion of patients did not receive the current standard of care. This is mainly due to the long inclusion period and the fact that not all patients were managed in wards experienced in Hepatology. Third, the result was confirmed in patients with HBV infection, which needs further validation for other etiology.

## Conclusions

The present study confirmed HDL-C as an independent predictor of 6-week mortality in HBV-associated cirrhosis patients with acute gastrointestinal bleeding. The developed model predicts 6-week mortality with higher accuracy than the existing prognostic models. Moreover, it succeeded in stratifying patients into different risk groups, providing a basic assessment method to identify patients at high risk of death, making it possible to select those who should be managed in a more intensive way. Thus, further studies are warranted to validate its effectiveness as a predictor of bleeding in cirrhotic patients.

## Data Availability

All data generated or analysed during this study are included in this published article and its supplementary information files.

## Abbreviations

ALBI: Albumin-bilirubin score
ALB: Albumin
ALT: Alanine aminotransferase
AVB: Acute variceal bleeding
CI: Confidence intervals
ECG: Electrocardiogram
FIB: Fibrinogen
HBV: Hepatitis B virus
HCC: Hepatocellular carcinoma
HDL-C: High-density lipoprotein cholesterol
HGB: Hemoglobin
INR: International normalized ratio
IQRs: Interquartile ranges
LDL-C: Low-density lipoprotein cholesterol
MELD: Model of end-stage liver disease
Na: Serum sodium
NVB: Nonvariceal bleeding
OR: Odds ratio
PRBC: Packed red blood cell transfusions
Scr: Serum creatinine
TBIL: Total bilirubin
TIPS: Transjugular intrahepatic portosystemic shunt
WBC: Total leukocyte count

## References

[1] Global, regional, and national age-sex specific all-cause and cause-specific mortality for 240 causes of death, 1990–2013: a systematic analysis for the Global Burden of Disease Study 2013. Lancet. 2015; 385(9963):117–171. 10.1016/S0140-6736(14)61682-2 PMC4340604 25530442

[2] Hsu YC, Lin JT, Chen TT, Wu MS, Wu CY (2012). Long-term risk of recurrent peptic ulcer bleeding in patients with liver cirrhosis: a 10-year nationwide cohort study. Hepatology.

[3] A A, G I-S, J P, et al. Survival of patients with cirrhosis and acute peptic ulcer bleeding compared with variceal bleeding using current first-line therapies. Hepatology (Baltimore, Md). 2018;67(4):1458–1471. 10.1002/hep.29370 28714072

[4] Bambha K, Kim WR, Pedersen R, Bida JP, Kremers WK, Kamath PS (2008). Predictors of early re-bleeding and mortality after acute variceal haemorrhage in patients with cirrhosis. Gut.

[5] Goulis J, Armonis A, Patch D, Sabin C, Greenslade L, Burroughs AK (1998). Bacterial infection is independently associated with failure to control bleeding in cirrhotic patients with gastrointestinal hemorrhage. Hepatology.

[6] Pugh RN, Murray-Lyon IM, Dawson JL, Pietroni MC, Williams R (1973). Transection of the oesophagus for bleeding oesophageal varices. Br J Surg.

[7] Wiesner R, Edwards E, Freeman R (2003). Model for end-stage liver disease (MELD) and allocation of donor livers. Gastroenterology.

[8] Johnson PJ, Berhane S, Kagebayashi C (2015). Assessment of liver function in patients with hepatocellular carcinoma: a new evidence-based approach-the ALBI grade. J Clin Oncol.

[9] D'Amico G, De Franchis R (2003). Upper digestive bleeding in cirrhosis. Post-therapeutic outcome and prognostic indicators. Hepatology.

[10] Augustin S, Altamirano J, González A (2011). Effectiveness of combined pharmacologic and ligation therapy in high-risk patients with acute esophageal variceal bleeding. Am J Gastroenterol.

[11] Saltzman JR, Tabak YP, Hyett BH, Sun X, Travis AC, Johannes RS (2011). A simple risk score accurately predicts in-hospital mortality, length of stay, and cost in acute upper GI bleeding. Gastrointest Endosc.

[12] Blatchford O, Murray WR, Blatchford M (2000). A risk score to predict need for treatment for upper-gastrointestinal haemorrhage. Lancet.

[13] Perez-Matos MC, Sandhu B, Bonder A, Jiang ZG (2019). Lipoprotein metabolism in liver diseases. Curr Opin Lipidol.

[14] Ooi K, Shiraki K, Sakurai Y, Morishita Y, Nobori T (2005). Clinical significance of abnormal lipoprotein patterns in liver diseases. Int J Mol Med.

[15] Etogo-Asse FE, Vincent RP, Hughes SA (2012). High density lipoprotein in patients with liver failure; relation to sepsis, adrenal function and outcome of illness. Liver Int.

[16] Habib A, Mihas AA, Abou-Assi SG (2005). High-density lipoprotein cholesterol as an indicator of liver function and prognosis in noncholestatic cirrhotics. Clin Gastroenterol Hepatol.

[17] Pirillo A, Catapano AL, Norata GD (2015). HDL in infectious diseases and sepsis. Handb Exp Pharmacol.

[18] Zambruni A, Di Micoli A, Lubisco A, Domenicali M, Trevisani F, Bernardi M (2007). QT interval correction in patients with cirrhosis. J Cardiovasc Electrophysiol.

[19] Hanley JA, McNeil BJ (1983). A method of comparing the areas under receiver operating characteristic curves derived from the same cases. Radiology.

[20] Fluss R, Faraggi D, Reiser B (2005). Estimation of the Youden Index and its associated cutoff point. Biometrical J Biometrische Zeitschrift.

[21] Lemeshow S, Hosmer DW (1982). A review of goodness of fit statistics for use in the development of logistic regression models. Am J Epidemiol.

[22] El-Serag HB, Everhart JE (2000). Improved survival after variceal hemorrhage over an 11-year period in the Department of Veterans Affairs. Am J Gastroenterol.

[23] Bassendine MF, Sheridan DA, Bridge SH, Felmlee DJ, Neely RD (2013). Lipids and HCV. Semin Immunopathol.

[24] Ramcharran D, Wahed AS, Conjeevaram HS (2011). Serum lipids and their associations with viral levels and liver disease severity in a treatment-naïve chronic hepatitis C type 1-infected cohort. J Viral Hepat.

[25] MacCallum PK, Cooper JA, Martin J, Howarth DJ, Meade TW, Miller GJ. Haemostatic and lipid determinants of prothrombin fragment F1.2 and D-dimer in plasma. Thrombosis and haemostasis. 2000;83(3):421–426. 10744148

